# Numerical and Machine Learning Analysis of the Parameters Affecting the Regionally Delivered Nasal Dose of Nano- and Micro-Sized Aerosolized Drugs

**DOI:** 10.3390/ph16010081

**Published:** 2023-01-06

**Authors:** Ali Farnoud, Hesam Tofighian, Ingo Baumann, Kaveh Ahookhosh, Oveis Pourmehran, Xinguang Cui, Vincent Heuveline, Chen Song, Sarah Vreugde, Peter-John Wormald, Michael P. Menden, Otmar Schmid

**Affiliations:** 1 Computational Health Center, Helmholtz Munich, 85764 Neuherberg, Germany; 2 Comprehensive Pneumology Center (CPC-M), Member of the German Center for Lung Research (DZL), 81377 Munich, Germany; 3 Institute of Lung Health and Immunity, Helmholtz Zentrum München–German Research Center for Environmental Health, 85764 Neuherberg, Germany; 4 Department of Mechanical Engineering, Amirkabir University of Technology, Tehran 1591634311, Iran; 5 Department of Otorhinolaryngology, Head and Neck Surgery, Medical Center of Heidelberg University, 69120 Heidelberg, Germany; 6 Biomedical MRI and MoSAIC, Department of Imaging and Pathology, KU Leuven, 3000 Leuven, Belgium; 7 Department of Otolaryngology, Head and Neck Surgery, Adelaide Medical School, The University of Adelaide, Adelaide 5011, Australia; 8 School of Mechanical Engineering, The University of Adelaide, Adelaide 5005, Australia; 9 School of Aerospace Engineering, Huazhong University of Science and Technology, Wuhan 430074, China; 10 Engineering Mathematics and Computing Lab (EMCL), Heidelberg University, 69120 Heidelberg, Germany; 11 Department of Biology, Ludwig-Maximilian University Munich, 82152 Planegg, Germany; 12 German Center for Diabetes Research (DZD e.V.), 85764 Neuherberg, Germany

**Keywords:** nasal drug delivery, machine learning, numerical modelling, nanodrug delivery, targeted drug delivery

## Abstract

The nasal epithelium is an important target for drug delivery to the nose and secondary organs such as the brain via the olfactory bulb. For both topical and brain delivery, the targeting of specific nasal regions such as the olfactory epithelium (brain) is essential, yet challenging. In this study, a numerical model was developed to predict the regional dose as mass per surface area (for an inhaled mass of 2.5 mg), which is the biologically most relevant dose metric for drug delivery in the respiratory system. The role of aerosol diameter (particle diameter: 1 nm to 30 µm) and inhalation flow rate (4, 15 and 30 L/min) in optimal drug delivery to the vestibule, nasal valve, olfactory and nasopharynx is assessed. To obtain the highest doses in the olfactory region, we suggest aerosols with a diameter of 20 µm and a medium inlet air flow rate of 15 L/min. High deposition on the olfactory epithelium was also observed for nanoparticles below 1 nm, as was high residence time (slow flow rate of 4 L/min), but the very low mass of 1 nm nanoparticles is prohibitive for most therapeutic applications. Moreover, high flow rates (30 L/min) and larger micro-aerosols lead to highest doses in the vestibule and nasal valve regions. On the other hand, the highest drug doses in the nasopharynx are observed for nano-aerosol (1 nm) and fine microparticles (1–20 µm) with a relatively weak dependence on flow rate. Furthermore, using the 45 different inhalation scenarios generated by numerical models, different machine learning models with five-fold cross-validation are trained to predict the delivered dose and avoid partial differential equation solvers for future predictions. Random forest and gradient boosting models resulted in R^2^ scores of 0.89 and 0.96, respectively. The aerosol diameter and region of interest are the most important features affecting delivered dose, with an approximate importance of 42% and 47%, respectively.

## 1. Introduction

The human extrathoracic region, including the nasal and oral cavities, pharynx, and larynx, acts as a filter for inhaled particulates, mainly micron-sized aerosols (1 to 80 µm), and it plays an important role in the management of harmful aerosols in the human respiratory system [1]. The nasal cavity is the primary line of defense against particulate pollutants penetrating deep into the lungs. However, this filtering process hinders the delivery of therapeutic aerosols to the desired region in the upper and lower respiratory tracts. The nasal airway is a potential route for drug administration due to its rich vascular plexus [1]. Recent advances in nano- and micro-scale therapeutics have shed new insight into the treatment of various disorders [2]. Consequently, targeted drug delivery through nasal and oral routes has gained traction in recent years [3]; however, due to the geometrical complexity of nasal and oral airways, a deep understanding of aerosol deposition patterns and the mechanisms underlying the dispersion of aerosols in these regions is still needed to be developed.

Over the past decades, there has been an increasing amount of literature describing the role of dispersion and deposition of nano- and micro-aerosols in the human nasal cavity, both experimentally and numerically. In 1988, Cheng et al. [4] measured the deposition of ultrafine aerosols ranging from 3.6 to 150 nm using a clear polyester resin cast for the upper airways of a human adult. In 2004, Kelly et al. [5,6] investigated the effect of surface roughness on the deposition of nano- and micro-aerosols. Liu et al. [7] numerically and experimentally studied aerosol deposition in Carleton-Civic standardized geometry where aerosol diameters ranged from 1.71 to 9.14 µm carried by high inhalation flow rates (30 to 90 L/min) and found that inertial impaction was the dominant aerosol deposition mechanism for the dispersion of micro-aerosols in turbulent flow conditions. In a similar investigation, Schroeter et al. [8] experimentally and numerically investigated the regional deposition of monodisperse micro-aerosols in the range of 2.6 and 14.3 µm and airflow rates of 15 L/min in a sectional nasal cast. Their results showed maximum values of 15%, 7%, and 12% in the anterior turbinates, olfactory, and turbinates regions, respectively. Moreover, Basu et al. [9] suggested new methods to enhance the deposition efficiency of micro-aerosols and used 3D printed replicas to validate the numerical results. Several researchers have studied the micro-aerosols delivery into different regions of the nasal cavity such as maxillary sinuses, olfactory regions and turbinates [10,11,12,13,14,15,16,17,18]. In recent years, different methods such as imposing conditions of pulsatile airflow [12,13,19], acoustic waves superposition [10,11,20], and swirling flow [14] at the nostrils have been studied.

Recently, more studies used numerical and experimental methods to understand the deposition patterns of nano- and micro-aerosols in the upper respiratory system [21,22,23,24,25,26]. Shi et al. [21] simulated the deposition efficiency of nano-aerosols in diameter ranges of 1 to 150 nm carried by relatively low airflow rates of 7.5 to 20 L/min in a realistic nasal airway model. They observed high deposition efficiencies at smaller nano-aerosols. In their comparative study, Garcia et al. [27] simulated the deposition of nano-aerosols in the human olfactory epithelium with low inhalation flow rates, and found that the total deposition rate was lower in the human olfactory region than in rats under similar conditions. In a previous study [16], we showed a bi-directional drug delivery technique with pulsating and non-pulsating inlet airflow conditions to assess deposition efficiencies in the olfactory region. They reported that the deposition efficiency in the olfactory region without and with pulsating airflow was 0.48% and 0.12%, respectively. Additionally, they showed that imposing pulsatile inlet airflow during bi-directional drug delivery reduced the non-uniformity of right–left deposition in both the cavity (from 1.77- to 1.33-fold) and the olfactory region (from 624- to 53.2-fold). Inthavong et al. [28] analyzed the airflow in six nasal airway replicas of different subjects to study the subject-dependent variations in the numerical investigation; however, the results suggest consistent flow patterns in the nasal airways. Kiaee et al. [29] studied the regional deposition in seven computed tomography (CT)-based nasal cavity replicas to evaluate the effects of parameters such as aerosol diameter, spray cone angle, and aerosol injection speed, which showed high variability in olfactory depositions for the airway replicas. In 2020, Farnoud et al. [12] used a dynamic large eddy simulation (LES) model to simulate airflow and micro-aerosols dispersion and deposition during bi-directional pulsatile aerosol delivery with clockwise 45° and 90° nosepieces. Their results showed that drug delivery with pulsating inlet airflow resulted in a more uniform deposition pattern compared to non-pulsating flow in the nasal airways. In another study, Farnoud et al. [13] studied the effect of flow rate and micro-aerosol size on deposition efficiencies in the nasal cavity, specifically the maxillary sinuses. They observed enhancement in deposition efficiency by applying a bi-directional delivery method. Moreover, they reported enhancement of deposition by increasing the size of the micro-aerosols and the flow rate.

Despite numerous efforts in recent decades to gain insight into the dispersion and deposition mechanism of nano- and micro-aerosols in different regions of the nasal cavity, it is essential to investigate the behavior of aerosols across a wide range of sizes and inhalation rates. More importantly, delivered regional dose is better calculated as mass per surface area, which is biologically the most meaningful and effective metric in respiratory therapeutic aerosol delivery [30,31]. Therefore, in this study, we aimed to conduct a comprehensive numerical study on local aerosol deposition quantified by mass per surface area for a wide range of aerosol sizes (fifteen different diameters between 1 nm and 30 µm), under different inhalation flow rates (laminar, transitional, turbulent flow conditions) using the local dynamic LES model, which can capture laminar-transitional-turbulent flows [32]. Different machine learning (ML) models with five-fold cross-validation were built on the generated data using computational fluid dynamics (CFD) and the two best performing models of gradient boosting and random forest were used for the real-time prediction of regional dose for nasal drug delivery. Furthermore, the importance of variables on the regional nasal dose were assessed and discussed.

## 2. Results

Nano- and micro-aerosols with a wide range of diameters, ranging from 1 nm to 30 µm, were injected from both nostrils, and inlet airflow rates (from 4 to 30 L/min) were implemented at the inlet, which cover laminar, transitional, and turbulent flow conditions in different regions of the airway. The local dynamic LES model was coupled with the Lagrangian Intermediate library in OpenFOAM 3.0.0 (see Section 6), which was able to simulate the aerosol motion in laminar-transitional-turbulent flow conditions.

### 2.1. Mesh Quality

**Figure 1:**
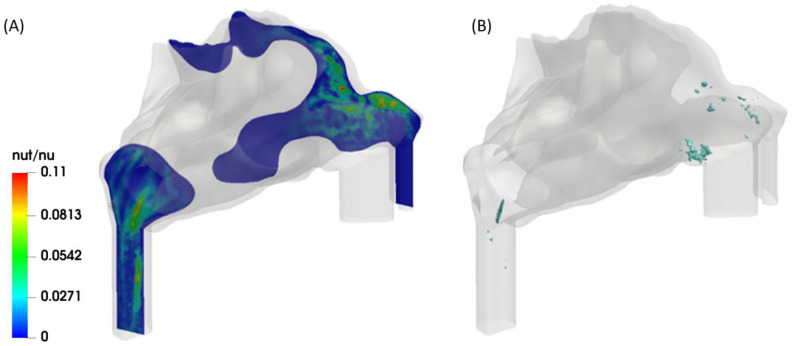
The viscosity ratio as indicator to ensure the mesh quality for LES analysis (nut/nu, where nut and nu are turbulent and molecular kinematic viscosities, respectively). (**A**): cross-section colored by the viscosity ratio. (**B**): iso-surface of the viscosity ratio of 0.1.

The resolution of the computational grid has a strong impact on the accuracy of LES results. One method to determine the resolution quality of an LES simulation is to examine the viscosity ratio on a sub-grid scale (nut) and molecular viscosity (nu). The lower the viscosity ratio, the more the turbulence is resolved and, consequently, the precision of the simulation is higher due to the higher resolution quality. To ensure that the grid resolution is fine enough, Pelmard et al. [33] suggested that the viscosity ratio should be lower than 0.3. For this reason, the viscosity ratios are shown on a cross-section inside the domain that contains the most turbulent region of the airway. Moreover, the viscosity ratio iso-surface is shown in Figure 1B. What stands out from Figure 1A is that the ratio is higher than 0.3 in the whole domain, which means that the grid is fine enough for the present simulation. Additionally, the iso-surface shows that the greatest amount is located in the vestibule and nasal valve (anterior region) and the nasopharynx (posterior region). The final mesh with the required resolution has approximately 4 million cells.

### 2.2. Validation of the Model

**Figure 2:**
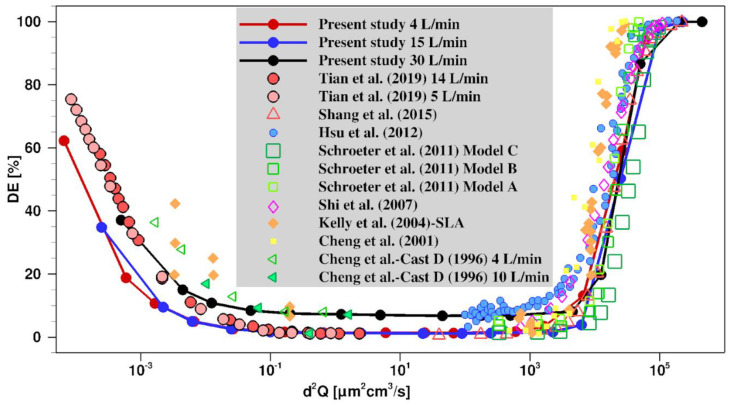
Comparison of the aerosol deposition efficiency (DE) inside the present nasal cavity and the other realistic nasal cavities reported in the literature scaled by Stokes number for unity density aerosol (*d*^2^*Q*) [15,34,35,36,37,38,39].

To validate aerosol deposition in the realistic nasal airway cavity, the aerosol deposition efficiency was calculated on the wall of the airway and compared with the experimental and numerical results available in the literature for nano- and microsized aerosol in the human nasal airway [15,34,35,36,37,38,39]. Figure 2 shows the comparison of the CFD-simulated deposition efficiency (DE) for nano- and micro-aerosols in the present realistic nasal airway and the DEs from empirical in vitro cast and numerical studies in the literature [15,34,35,36,37,38,39]. The data are presented as a function of the Stokes number for unity density aerosol (*d*^2^*Q*), which is the characteristic parameter for aerosol impaction. Considering the difference in geometry of nasal cavities, there is good agreement between the calculated results from the present study and literature data in both the nano- and micro-site range.

### 2.3. Airflow during Bilateral Aerosol Delivery

**Figure 3:**
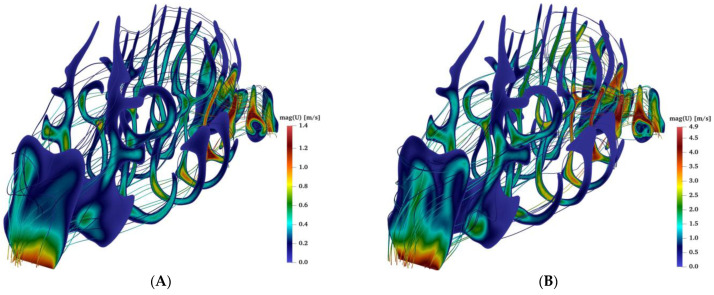

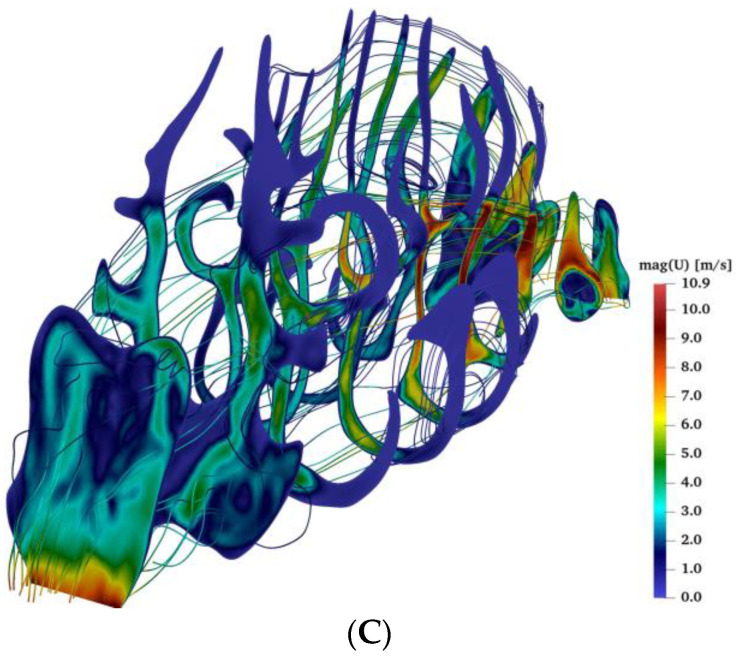
The velocity contours and streamlines for the nasal airflow at different flow rates of 4 (**A**), 15 (**B**), and 30 L/min (**C**).

Airflow with aerosol enters the nose from both nostrils (bilateral), and after passing through the complex nasal geometry it reaches the nasopharynx region and exits the nasal passage. Due to the presence of several curvatures and bends in the nasal geometry, each region has its special flow characterization. For example, in the vestibule region (near the nostrils) recirculation of the flow is observed. In the connected (nasal valve) region and the nasopharynx the flow velocities reach the highest values (see Figure 3). Depending on the size of the aerosols (nano- or micro-aerosols), diffusion or inertial impaction plays a crucial role in their deposition mechanisms and the air velocity and flow complexity, such as the creation of recirculation zones or swirling flows, directly influence the aerosol dispersion and deposition.

### 2.4. Regional Delivered Dose

**Figure 4:**
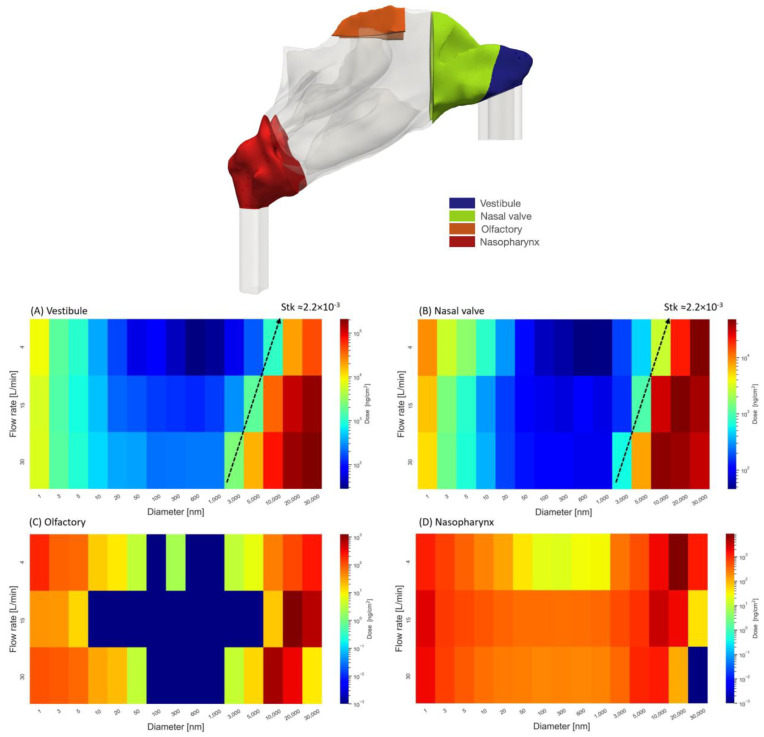
The locations of the vestibule, nasal valve, and nasopharynx are visualized in the upper panel. In four regions of the vestibule (**A**), nasal valve (**B**), the olfactory epithelium (**C**), and the nasopharynx (**D**), the corresponding delivered dose in the form of mass per square centimeter is calculated for three airflow rates of 4, 15, 30 L/min and aerosols with diameters between 1 nm and 30 µm.

Hot spot regions of aerosol deposition on the nasal epithelium can be identified from the surface area-normalized deposited dose, i.e., as the deposited aerosol dose (mass) per surface area. The spatially resolved, delivered doses are calculated for aerosols between 1 nm and 20 µm (15 different diameters) carried by an airflow with inlet flow rates of 4, 15 and 30 L/min. The aerosol velocities at the inlets (nostrils) are assumed to be identical to that of the airflows. Typical nasal spray conditions are considered and 100,000 computational particles (parcels) of each size group are injected with a total injection mass of 2.5 mg assuming unit density (water) aerosol. Figure 4 illustrates the deposited dose in four regions of the nasal cavity for different flow rates and aerosol sizes. Due to the large range of delivered doses and aerosol sizes both parameters are presented on a logarithmic scale. The highest area doses are observed in the 10–30 µm size range, and these maximum values decrease from 2.08 × 10^5^ ng/cm^2^ in the vestibule region to 4.35 × 10^4^, 8.79 × 10^3^, and 1.24 × 10^3^ ng/cm^2^ in the nasal valve, nasopharynx and olfactory bulb region, respectively. On the other hand, for diffusion-dominated nano-aerosols, somewhat lower maximum deposition values of 8.99 × 10^3^, 8.27 × 10^3^, 2.32 × 10^3^, and 1.99 × 10^2^ ng/cm^2^ are calculated for the nasal valve, vestibule, nasopharynx, and olfactory bulb region, respectively. Since diffusion effects are the largest for small aerosol size and long residence time, most of these maximum doses are observed for the smallest aerosol size (1 nm) and the smallest flow rate (4 L/min).

The deposited dose for diffusion-dominated nano-aerosol decreases as the nano-aerosol size increases from 1 nm. The Brownian motion influences the dispersion of nano-aerosols with diameters smaller than 20 nm, and as a general pattern, for these aerosols, higher deposited doses are observed in all nasal regions. For nano-aerosols larger than 20 nm, very low deposited doses are observed in the vestibule, nasal valve, and olfactory region. With regard to micro-aerosols, as the Stokes numbers increase, the chance of aerosols escaping from the streamlines in sudden changes of flow direction increases; hence, higher Stokes numbers lead to higher deposited doses in those regions except for posterior regions of the nasal cavity (nasopharynx and olfactory region). Large aerosols (30 µm) carried by high airflow rates (30 L/min) tend to deposit in the anterior parts of the nasal cavity, and as a result less aerosols could deposit in the posterior regions of the airway.

In Figure 4A, an iso-line represents a Stokes number of 2.2 × 10^−3^. Here, the Stokes number was defined by the characteristic length equal to nasal airway length of 10 cm and gas velocity equal to inlet velocity. This line separates the higher and lower ranges of micro-aerosols deposition rates. Micro-aerosols with a Stokes number higher than this value can escape from the flow streamlines because of their inertia or turbulence dispersion in the case where turbulence exists (higher flow rates). Figure 4B depicts the delivered dose to the nasal valve. In general, fine nano-aerosols, especially large micro-aerosols, lead to high deposition in the nasal valve. Similar to Figure 4A, an iso-line of Stokes equal to 2.2 × 10^−3^ is separating the low and high dose conditions.

Optimal drug delivery to the olfactory epithelium plays a crucial role in drug delivery from the nose to the brain [16]; therefore, we discuss this issue in more detail (see Figure 4C). For low flow rates (4 L/min), aerosol sizes smaller than 20 nm and larger than 10 µm result in the highest delivered doses of 1.99 × 10^2^ and 1.24 × 10^3^ ng/cm^2^. The maximum delivered dose occurs when aerosols with diameters of 1 nm and 20 µm are used. For moderate and high flow rates (15 and 30 L/min), a similar pattern was observed with the maximum delivered dose associated with aerosols diameters of 20 and 10 µm, respectively.

As aerosols move forward from the vestibule towards the nasopharynx (anterior to posterior regions), the overall delivered dose reduces, which could be noticed in Figure 4 (compare values in the legends). Aerosols with a high chance of deposition, micro-aerosols with a large Stokes number, have been deposited before reaching the posterior regions (nasopharynx). It is evident that for very high flow rates and large micro-aerosols, no aerosol could reach the nasopharynx since they all deposit upstream (mainly in the nasal valve and vestibule). The overall heatmap of the dose delivered to the nasopharynx has lower dose rates and a relatively uniform pattern.

### 2.5. Dose Prediction Using Machine Learning

**Figure 5:**
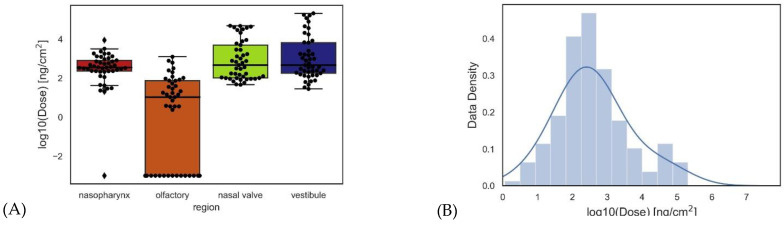
The distribution of the data extracted from the numerical analysis is represented as boxplots (**A**) and histograms (**B**).

This study predicts dose for three different flow rates, fifteen diameters, and four different regions, resulting in 180 data points for the delivered dose. The distribution of the dose in different regions of the nasal cavity is illustrated using boxplots (see Figure 5). For better visualization, the results are depicted on a logarithmic scale. The data distribution in all regions is similar except for the olfactory region where the median is significantly lower than in other regions (t test performed), which shows that the dose in the olfactory region is much lower than in other regions of the airway. Moreover, the data density is shown on a logarithmic scale, which has Gaussian distribution (Figure 5).

**Figure 6:**
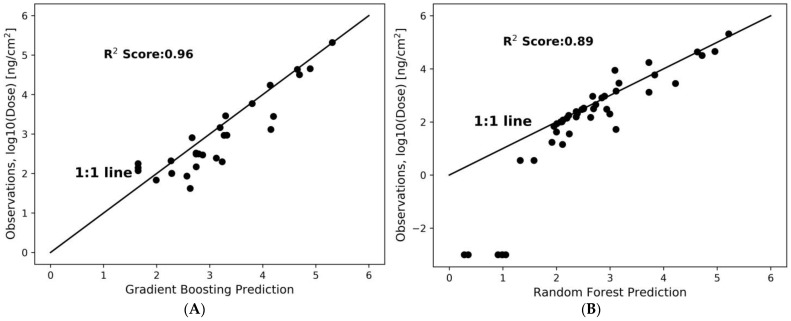
Prediction of the regionally delivered dose using the gradient boosting (**A**) and random forest (**B**) models compared to CFD-driven results (observations). Blue dots show the CFD-driven and ML-driven dose predictions and the black line is the identity line (1:1 line).

ML models are developed using these data to predict the dose in the different regions of the human nasal cavity. Several ML models including k-nearest neighbors (KNN), lasso, elastic net, random forest, and gradient boosting are trained in the present dataset with five-fold cross-validation, and the two models of gradient boosting and random forest, which lead to the highest R^2^ scores, are shown in Figure 6. Figure 6 shows the CFD-driven dose (observations) versus predicted dose based on ML models and, for better visualization, plots are visualized on logarithmic scales. The R^2^ scores of gradient boosting and random forest models are 0.96 and 0.89, respectively.

**Figure 7:**
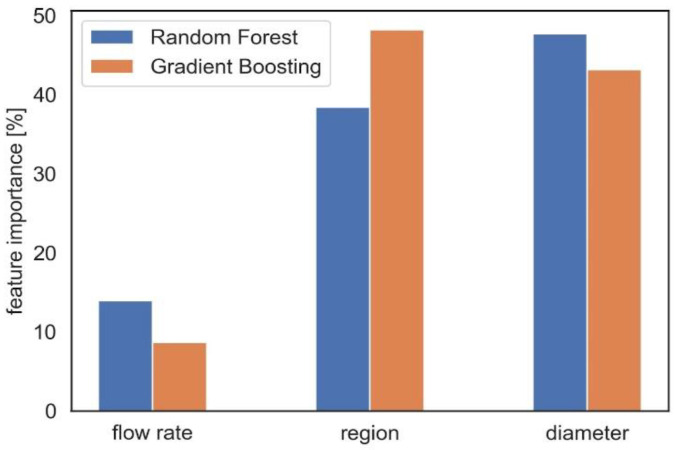
The flow rate, the region of interest, and the aerosol diameter are factors that affect the regional deposited dose, and the random forest and gradient boosting models are used to determine their importance in predicting the dose.

Here, the focus is on three main features, namely, flow rate, aerosol diameter, and region of interest. An analysis using random forest and gradient boosting models is performed to assess the influence of these covariates/features on the deposited dose. By applying feature importance analysis using the gradient boosting model, we observe that diameter and region of interest are the most important features affecting delivered doses, with an approximate importance of 42% and 47%, respectively (Figure 7). The deposition is influenced by airflow rate to a degree of approximately 8%.

## 3. Discussion

This study aims to investigate the delivered dose in different regions of the nasal airway. In previous numerical studies [12,19], regional deposition efficiency of inhaled medication was reported with percentage of deposited drug; however, in this study, dose as mass per surface area was considered, which is the biologically most relevant dose metric for respiratory delivery of aerosolized drugs and toxins [30,31,40]. Three different inlet flow rates and fifteen different aerosol diameters including a range of nano- and micro-aerosols are considered in the simulations to predict the deposited dose in four different regions. The results reveal the most optimal drug delivery conditions (aerosol diameter and inhalation flow rate) into the olfactory epithelium, nasopharynx, nasal valve and vestibule. In total, 180 data points are generated and different ML models are implemented to have a tool for the real-time prediction of dose as mass per surface area in different regions of the nasal cavity without requirement to solve partial differential equations. Moreover, the analysis of the importance of features affecting the delivered dose showed that aerosol diameter plays a much more important role in deposition than the flow rate, which is in good agreement with the literature [5,36]. For practical purposes, this is an important aspect, since the aerosol size is readily controlled by the design of the medical device, while inhalation flow rate is more complicated to control in clinical settings.

In general, for the nano-sized aerosols (here: smaller than 300–600 nm, depending on flow rate and nasal region) the deposited dose increases with decreasing aerosol size, while for micro-aerosols the deposited dose increases with increasing aerosol diameter. This is consistent with diffusion and impaction being the dominant mechanisms of aerosol deposition in the nano- and micro-aerosol range in the entire respiratory tract (including the nose), respectively [41,42]. Nano-aerosol with diameters larger than 20 nm lead to low doses in all regions, and for mass doses per surface area, which are similar to the maximum deposited dose obtained with micro-aerosols, one would need to apply 1 nm particles. Inhalation of 2.5 mg (as assumed here) would require generation of 5 × 10^19^ particles within a few seconds (inhalation time). To the best of our knowledge, there is currently no method available that can accomplish this using solid or liquid substances (only gas-to-particle conversion methods can accomplish this) [43]. Thus, microparticles should be used for nasal drug delivery. However, for future possible drug delivery device design, we performed a comprehensive study to design optimal drug delivery with nano-aerosols as well.

As a result of the centrifugal forces, radial pressure gradients occur in the nasal valve region driving the airflow towards the outer side (wall). Microparticles with high Stokes numbers do not easily follow the streamlines of the airflow after the bends in regions such as the vestibule, nasal valve, and nasopharynx, leading to higher deposition rates in these regions [13]. In our previous work on the deposition pattern of the micro-aerosols in this nasal cavity [12,13] we showed an intense (hot spot) deposition pattern in the anterior and posterior regions of the nasal cavity. Furthermore, it was found that as the aerosol size increases, more aerosols tend to deposit in the lower part of the nasal respiratory zone [12]. As expected for impaction-driven aerosol deposition, we observe that the deposited dose of micro-aerosols is positively correlated with Stokes number, i.e., higher Stokes number leads to higher deposited doses. To obtain the highest doses in the olfactory region, we suggest aerosols with a diameter of 20 µm and inlet (inhalation) air flow rate of 15 L/min. Moreover, our results show that high flow rates and larger micro-aerosols lead to highest doses in the vestibule and nasal valve regions. Consequently, fine micro aerosols with low inlet airflow rates cause higher doses in the nasopharynx. For inlet airflow rates of 15 and 30 L/min carrying large aerosols with diameters of 30 and 20 µm, respectively, rarely was the drug able to reach the nasopharynx since most of the aerosols were already deposited in the anterior parts due to the high inertial impaction.

The limitations of this study include the absence of interindividual and intraindividual variability of geometrical features and numerical assumptions for solving the PDEs. For future work, we suggest a study on nasal geometries derived from CT images of several patients to build a population for a more comprehensive study.

## 4. Materials and Methods

### 4.1. Geometry

**Figure 8:**
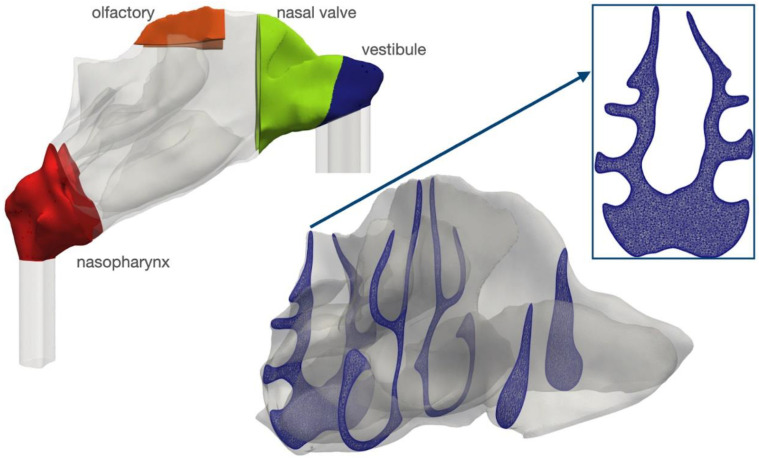
The anatomy of the nasal cavity with vestibule, nasal valve, olfactory, and nasopharynx is colored in the left figure. A detailed cross-section of the mesh inside the nasal cavity is shown, along with a closeup view of one of the most complex cross-sections inside the nasopharynx (right figure).

In this study, a CT-based geometry of the nasal airway of an 80-year-old patient was reconstructed using 3DSlicer software under the supervision of clinical experts from Heidelberg University Hospital. The CT slice thickness of the CT images were 1 mm and the resolution of the images were 2.32 pixels per mm. This specific nasal airway had a length of 10 cm and a slight septum deviation to the left side. Detailed information on the images was provided in the previous investigation by the authors [13] in which bi-directional drug delivery was studied. Here, bilateral nasal drug delivery is the focus of the research. Airflow carrying the aerosolized drug enters from both nostrils and passes through different regions of the nasal airway and eventually exits the domain at the nasopharynx. As suggested in the literature [37], extension pipes are added to the inlet (entrance of nostrils) and outlet (exit of nasopharynx) to control the flow effects and create fully developed conditions at the entrance and exit of the modeling domain, which mimics a typical nasal drug delivery process. Figure 8 shows the geometry of the nasal cavity and specific regions in the nasal passage such as the vestibule, nasal valve, olfactory region and nasopharynx. Moreover, a view from the mesh of the current study is shown in Figure 8 with the advice of medical experts, and different regions of the nasal airway are labeled.

### 4.2. Numerical Modeling

In order to simulate the airflow and aerosol transport inside the nasal airway, local dynamic LES equations are coupled with the aerosol motion equations by considering one-way coupling, which ignores the impact of the aerosols on the gas-phase, as is the case for aerosol delivery to the nasal cavity [9,18,29,44]. In this study, airflow was simulated by solving the Eulerian equations of continuity and momentum. Simulation of aerosols was performed by solving Newton’s equation of motion numerically in a Lagrangian frame. The mathematical description of the fluid flow and aerosol equations are described in detail in the previous work of the authors [12,13,16,19]; therefore, in this work, the governing equations are not reported to avoid repetition.

To solve the one-way coupled Eulerian–Lagrangian aerosol-laden flow equations, the open-source software package of OpenFOAM (www.openfoam.org, accessed on 9 December 2022) was used. For the numerical solution of the airflow, the pimpleFoam algorithm was used, and to simulate the aerosol motion, the aerosol trajectory method from the Lagrangian Intermediate library was coupled with the pimpleFoam solver. LES with the local dynamic k-equation sub-grid scales (SGS) model was used for the simulation of the airflow. The present study covers a wide range of Reynolds numbers corresponding to the laminar-transitional-turbulent flows in a very complex geometry [13]; therefore, a local dynamic k-equation SGS model was used. The advantage of this model is that the coefficients (C_k_ and Cε) are chosen dynamically depending on time and space. The second-order accurate least-squares scheme was used for the discretization of the diffusion term. The “Gauss filteredLinear” scheme was employed for LES, which is a low-dissipation second-order central differencing method. The second-order “Gauss limitedLinear” scheme was utilized for the convection term of other scalar quantities. To discretize time, the second-order Upwind Euler (SOUE) scheme was used [45,46].

## 5. Conclusions

This study demonstrates that careful selection of aerosol diameter and flow rate allows for the effective targeting of aerosolized drugs to different regions of the nasal epithelium. Discarding nano-aerosol for therapeutic applications (no suitable aerosol generation method is currently available), the preferred aerosol diameter and airflow rate ranges for the four regions investigated here are 30 µm and 30 L/min (vestibule region), 30µm and 4 L/min, 20 µm and 15 L/min, 10 µm and 30 L/min (nasal valve), 20 µm and 15 L/min (olfactory bulb for nose-to-brain drug delivery), and 20 µm and 4 L/min (nasopharynx). Thus, depending on the (disease-specific) target region, the most suitable diameter and flow rate settings need to be selected for maximum dose delivery. With the CFD models, 180 data points for delivered dosage were generated to build different ML models with five-fold cross-validation, where the two best models were random forest and gradient boosting. Finally, the importance of the features, including the airflow rate, aerosol size, and region of deposition, is assessed based on the two ML models. According to gradient boosting, the diameter and region of interest contribute an importance of roughly 42% and 47% to the obtained delivered doses, respectively.

## 6. Code Availability

Source code for reproducibility of the figures and ML analysis is available at github: https://github.com/AFarnoud/respiratory_dose_calculator (accessed on 9 December 2022).

## Data Availability

Data is contained within the article.
